# A database of wild rice germplasm of *Oryza rufipogon* species complex from different agro-climatic zones of India

**DOI:** 10.1093/database/bay058

**Published:** 2018-07-02

**Authors:** Kabita Tripathy, Balwant Singh, Nisha Singh, Vandna Rai, Gauri Misra, Nagendra Kumar Singh

**Affiliations:** 1 ICAR, National Research Center on Plant Biotechnology, Pusa Campus, New Delhi 110012, India; 2 Amity Institute of Biotechnology, Amity University Campus, J-3 Block, Sector-125, Noida 201303, Uttar Pradesh, India

## Abstract

Rice is a staple food for the people of Asia that supplies more than 50% of the food energy globally. It is widely accepted that the crop domestication process has left behind substantial useful genetic diversity in their wild progenitor species that has huge potential for developing crop varieties with enhanced resistance to an array of biotic and abiotic stresses. In this context, *Oryza rufipogon*, *Oryza nivara* and their intermediate types wild rice germplasm/s collected from diverse agro-climatic regions would provide a rich repository of genes and alleles that could be utilized for rice improvement using genomics-assisted breeding. Here we present a database of detailed information on 614 such diverse wild rice accessions collected from different agro-climatic zones of India, including 46 different morphological descriptors, complete passport data and DNA fingerprints. The information has been stored in a web-based database entitled ‘Indian Wild Rice (IWR) Database’. The information provided in the IWR Database will be useful for the rice geneticists and breeders for improvement of rice cultivars for yield, quality and resilience to climate change.

Database URL: http://nksingh.nationalprof.in: 8080/iwrdb/index.jsp

## Introduction

Rice (*Oryza sativa* L) is one of the most important primary food crops of the world in terms of both volume and value and is single largest source of energy for more than half of the world’s population. The wild relatives of rice are great reservoir of genetic diversity, which can be used to improve the produce quality and quantity of rice (1, 2). They have survived for thousands of years in the nature, therefore must possess large repertoire of genes for resistance to various diseases and pests. The wild rice is also adapted to extreme habitats e.g. flood prone, drought prone, saline and acidic soil conditions and therefore must also have genes conferring tolerance to these extreme environmental conditions. Genes from wild rice have already made significant impact on rice improvement program. Examples of wild rice genes introgressed into cultivated rice include resistance to grassy stunt virus, bacterial leaf blight and brown plant hopper, cytoplasmic male sterility and heat and drought related traits (3–5).

The genus *Oryza* consists of 24 wild and two cultivated species (6, http://www.gramene.org/ Release #39, TBD 2013). Rice is a major cereal where tremendous genotypic and phenotypic diversity exists and about 1 20 000 different accessions are reported (7). Number of rice germplasm belonging to Aus, Indica, Japonica, Aromatic and Deep Water cultivar groups and their landraces are conserved in global gene banks (1, 3, 8, 9). International Rice Genebank Collection Information System (IRGCIS) shows that of the total 4645 accessions of wild rice species in the International Rice Research Institute (IRRI) Gene Bank, 838 accessions are of Indian origin (http://www.irgcis.irri.org/). Indian gene bank at the National Bureau of Plant Genetic Resources (NBPGR) has 307 accessions of *Oryza rufipogon*, 726 accessions of *Oryza nivara* (http://www.nbpgr.ernet.in:8080/PGRPortal/ as on 14 March 2018). The wild rice species in IRRI collection show tremendous diversity in morphological and agronomic traits such as plant height, days to flowering, growth habit, panicle structure, leaf angle, culm number, seed characteristics and adaptation to different habitats. Similarly a high level of genetic diversity is also reported for Indian wild rice (IWR) (10). After decoding the rice genome, hundreds of genes of agronomic importance have been cloned and validated for their function through genetic transformation. These include *Rc* gene for red pericarp (11), *CKX1* gene for high grain number (12), *SKC1* gene for salt tolerance (13), *GS3* gene for grain length (14), *BADH2* gene for fragrance of rice (15), *GBSS1* gene for grain quality (16), *Sub1* gene for submergence tolerance (17), *Xa21* genes for bacterial leaf blight resistance (18), *Pi1*, *Pi54* gene for blast resistance (19, 20) and so on. Each of these genes must have several alternative forms or alleles due to accumulation of spontaneous mutations and natural selection over thousands of years, particularly in the wild rice germplasm. To intensify the search for new useful genes and their alleles the information on wild rice accessions will be crucial.

India is divided into 15 agro-climatic zones: Western Himalyan, Eastern Himalyan, Lower Gangetic Plains, Middle Gangetic Plains, Upper Gangetic Plains, Trans-Gangetic Plains, Eastern Plateau and Hills, Central Plateau and Hills, Western Plateau and Hills, Southern Plateau and Hills, East Coast Plains and Hills, West Coast Plains and Ghat, Gujarat Plains and Hills, Western Dry Region and The islands, each with distinct ecological conditions (21). Indo-Burma region is an important center of rice diversity where large numbers of wild rice grow in their natural habitats. The cultivated varieties of rice represent only a small fraction of this variability and have comparatively much narrow genetic base due to domestication and breeding bottlenecks. Wild rice resources are depleting at an alarming rate and genes that have evolved through millions of years of evolution are becoming extinct because of heavy population pressure, increasing urbanization and industrialization. Therefore, it is important to collect, conserve and characterize the IWR germplasm. A database of all the available information on wild rice will be of utmost necessity for effective sharing and utilization of this valuable genetic resource.

## Database construction and content

### Data source

Reference information and basic passport data of the Indian *O. rufipogon Griff. Species Complex* (ORSC) wild rice accessions were obtained from the original collection sites in 13 different states and union territories, namely Andaman and Nicobar Islands, Assam, Bihar, Chhattisgarh, Goa, Gujarat, Himachal Pradesh, Madhya Pradesh, Maharashtra, Odisha, Uttar Pradesh, Uttarakhand and West Bengal. Till now we have collected 614 wild rice accessions from >500 remote villages in 64 districts (Table 1). We also obtained 15 accessions of *O. rufipogon* and 43 accessions of *O. nivara* from Gene Bank, NBPGR, New Delhi, India as reference for our study. For obtaining data on morphological characters, each germplasm was grown at IARI, New Delhi and data were recorded on 46 standard morphological descriptors (6, 22, 23). The morphological descriptors are divided into five groups viz. plant, culm, leaf, flower and seed. The IWR database also provides basic information on the 24 recognized wild rice species based on the literature (24). Total 418 accessions were characterized using *pSINE1*, SSR and SNP markers.

**Table 1.** Total wild rice accession collection from different states of India

Sr. no.	State and union territory	No. of accessions
1	Andaman and Nicobar Islands	10
2	Assam	25
3	Bihar	70
4	Chhattisgarh	51
5	Goa	29
6	Gujarat	54
7	Himachal Pradesh	48
8	Madhya Pradesh	10
9	Maharashtra	3
10	Odisha	94
11	Uttar Pradesh	191
12	Uttarakhand	9
13	West Bengal	20
Total		614

### Web interface, usage

**Figure 1.**
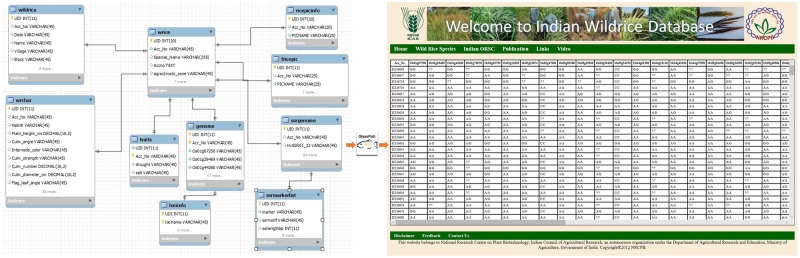
Schematic representation of the flow of data from the backend (MySQL) database to the frontend (JSP page) using middleware (Glassfish server) program.

The architecture of this online relational database is based on a ‘three-tier’ system, including client tier, middle tier and database. The Glassfish server is used here for connecting the client tier with the IWR database. The database was designed using MySQL 5.5. The client tier was created using Java server pages CSS 5.0 (Figure 1). The information has been stored in a web-enabled database entitled ‘Indian Wild Rice (IWR) Database’, is hosted at National Research Center on Plant Biotechnology, New Delhi, India.

**Figure 2.**
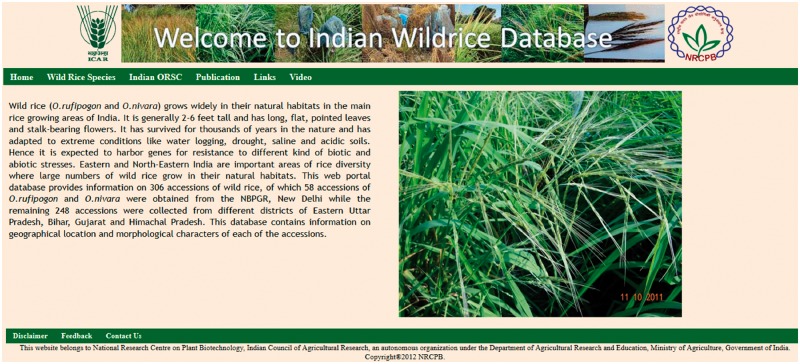
Home Page of Database showing a summary and feature tabs.

IWR Database has six different tabs to show the information on wild rice accessions (Figure 2).

**Figure 3.**
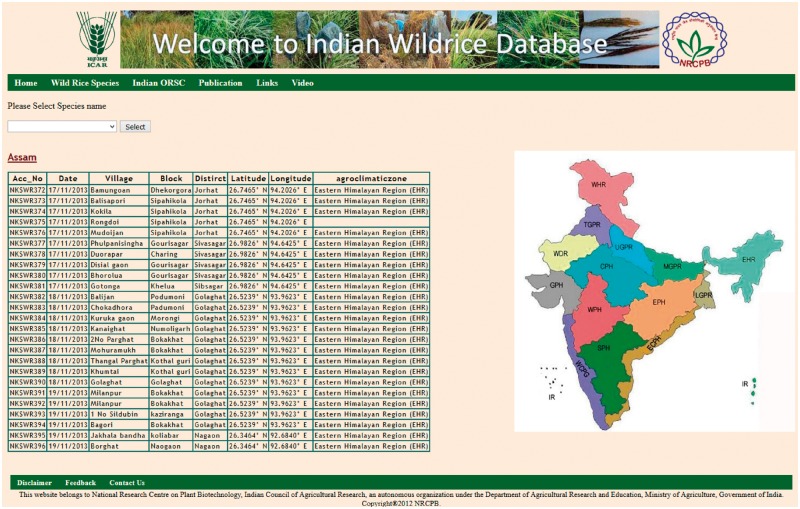
State wise collection information with latitude and longitude of places.

The ‘Wild Rice Species’ tab shows general information on 24 recognized wild rice species, including their genome type, geographical distribution, habitat, morphological characters and specific trait values. The ‘Indian ORSC’ tab contains information on our recently collected IWR accessions, which can be accessed freely online. This tab is further divided into five parts in the next page, by the species name, state name, accession number, SNP score and SSR score. The ‘Species’ tab has three options *O. nivara*, *O. rufipogon* and intermediate (*O. spontanea*) type. The ‘State’ tab contains the names of different states of India situated in different agro-climatic zone, from where the wild rice germplasm was collected (Figure 3).

**Figure 4.**
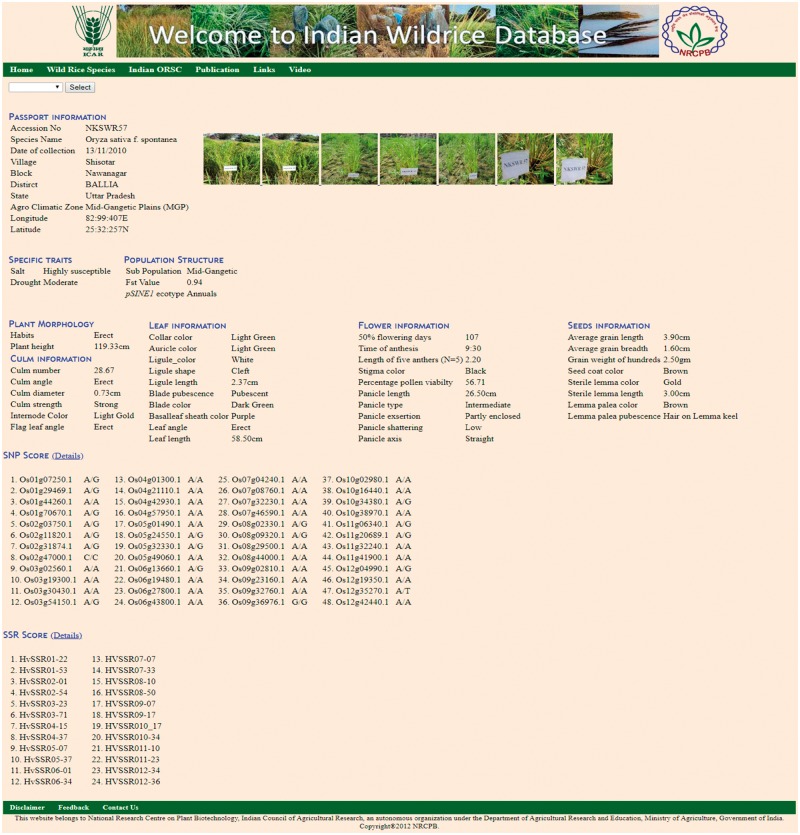
Accession page showing details of morphological characters with photograph and passport data.

The ‘Accession’ tab allows the user to choose the accession number to see information on 46 morphological descriptors sub-divided into five groups, namely Plant morphology, Culm information, Leaf information, Flower information and Seed information (Table 2, Figure 4), passport data and collection site with relevant photographs and videos. This tab also contains specific useful trait information on drought, salt tolerance and flood tolerance. This tab also contains molecular marker genotyping scores of the accessions based on 7 *pSINE1*, 48-plex GoldenGate SNP assay, 24 HvSSR markers. The ‘SNP score’ tab contains information genome wide 48 SNP markers for each accession. Similarly ‘SSR score’ tab contains 24 HvSSR markers information for each accession.

**Table 2.** Total 46 morphological characters list

S. no.	Name	S. no.	Name
1	Habits	24	Panicle axis
2	Plant height	25	Awning
3	Culm angle	26	Awn color
4	Internode color	27	Apiculus color
5	Culm strength	28	Awn length
6	Culm number	29	Leaf senescence
7	Culm diameter	30	Panicle shattering
8	Flag leaf angle	31	Stigma color
9	Blade pubescence	32	Time of anthesis
10	Blade color	33	Length of five anthers
11	Basal leaf sheath color	34	Percentage pollen viabilty
12	Leaf angle	35	Half flowering days
13	Leaf length	36	Lemma palea color
14	Leaf width	37	Lemma palea pubescence
15	Ligule color	38	Seed coat color
16	Ligule shape	39	Sterile lemma length
17	Collar color	40	Sterile lemma color
18	Auricle color	41	Average grain length
19	Ligule length	42	Average grain breadth
20	Panicle length	43	Ratio of grain length breadth
21	Panicle type	44	Grain weight of hundred
22	Secondary branching	45	Culm length
23	Panicle exsertion	46	Panicle threshability

### Database and website implementation

The IWR database provides information on 614 accessions of ORSC wild rice germplasm. Out of this 418 accessions were characterized in much detail and the remaining has only basic passport information. This database shows ORSC accessions classified based on their origin from diverse agro-climatic zones, morphological classification in to *O. nivara*, *O. rufipogon* and intermediate *O. sativa f. spontanea* types. This database also provides information on ecotype classification of the accessions in to annual, perennial, intermediate and unknown types based on *pSINE1* markers, sub-population information viz. Pro-Indica, Pro-Aus and Mid-Gangetic based on Fst values (10). This database also shows useful agronomic trait values of the accessions for flood, salinity and drought tolerance. This database will be useful for students, rice geneticists and breeders to know the process of rice domestication and improvement of rice cultivars for yield, quality and resilience to climate change.

## Conclusions and prospects

The IWR database is a primary source of information about large number of wild rice accessions collected from different agro-climatic zones of India. These wild rice accessions together with information on their salinity and drought tolerance phenotype will help rice geneticists and breeders to use these in genetic crossing programs to find and utilize agronomically useful genes. In order to utilize the collection efficiently, the accessions have been characterized based on morphological characters and molecular markers (10). Phenotypic screening of subsets of this collection has allowed identification of several accessions with high level of tolerance to drought, salinity and flooding (25, 26). More information on the trait related phenotypes of the accessions would be integrated with the IWR Database as and when it becomes available. Such information and the seeds of the accessions will be shared with rice geneticists and breeders according to the prevailing IPR and Biodiversity rules and guidelines. The creation of IWR database is the first step toward achieving these targets.

## Funding

We are thankful to Indian Council of Agricultural Research (ICAR) for financial support in the form of ‘ICAR-National Professor, B.P.Pal Chair’ project (Grant No. 27(13)/2009-HRD). Funding to pay the Open Access publication charges for this article was provided by ICAR.

*Conflict of interest*. None declared.
